# Surgical Management of Oral Cavity Cancer: Experience at a Tertiary Care Centre in Jamshedpur

**DOI:** 10.7759/cureus.51727

**Published:** 2024-01-05

**Authors:** Bijan K Saha, Sanghamitra Jena, Pankaj Singodia, Jayant K Lenka, Neetesh Sinha, Minakshi Mishra

**Affiliations:** 1 Surgical Oncology, Tata Main Hospital, Jamshedpur, IND; 2 Plastic and Reconstructive Surgery, Tata Main Hospital, Jamshedpur, IND; 3 Pathology, Tata Main Hospital, Jamshedpur, IND

**Keywords:** tobacco chewing, epidemology of oral cancer, recurrence, neck dissection, oral cavity cancer

## Abstract

Background

Cancer of the oral cavity is very common in Eastern India. This is due to the lack of awareness that chewing tobacco causes oral cancer. Because of poor economic condition and lack of access to healthcare, patients in this region often present at an advanced stage of the disease when they become symptomatic. A retrospective study was conducted at Tata Main Hospital, Jamshedpur, India, to know the epidemiology and recurrence of oral cavity cancer in this region.

Materials and methods

We conducted a retrospective study of oral cavity cancer patients operated at Tata Main Hospital, Jamshedpur, from January 2018 to June 2023. Data were collected from the surgical register, operation theatre notes, case sheets and hospital online data. The following parameters were observed in this study: a) age, b) gender, c) site of cancer, d) histology, e) stage of disease at presentation, f) type of neck dissection, g) margin status on the final histopathology report, h) node positivity, i) presence of perineural invasion or lymphovascular invasion and j) recurrence.

Results

A total of 218 patients were operated between January 2018 and June 2023. The most common site for oral cavity cancer was the buccal mucosa with the involvement of the lower alveolus (168 patients, 77.06%), followed by the tongue (27 patients, 12.38%). Two-hundred seventeen patients were diagnosed with squamous cell carcinoma (SCC), and one patient had epithelioid sarcoma on the biopsy report. The most common stage of presentation was stage IVa (180 patients, 82.56%), followed by stage III (16 patients, 7.34%). The most frequent neck dissection performed was modified radical neck dissection (MRND) sacrificing the sternocleidomastoid muscle (SCM) and preserving the internal juglar vein (IJV) and spinal accessory nerve (SAN) (176 patients, 80%). The margin was positive for 10 patients. Node positivity on the final histopathology report grouped according to the clinical stage are as follows: stage I (33.33%), stage II (60%), stage III (75%) and stage IV (86.67%). Similarly, the presence of lymphovascular or perineural invasion on the final histopathology report grouped according to the clinical stage is as follows: stage I (0%), stage II (20%), stage III (25%) and stage IV (55.55 %). Fifteen patients lost to follow-up. Recurrence was noted in 11 patients (5.04%). Patients presenting with stages I and II had no recurrence, whereas three out of 16 patients in stage III (1.1%) and eight out of 180 patients in stage IV (4.44%) had recurrence.

Conclusion

SCC is the most common type of oral cavity cancer in Eastern India. It is strongly related to tobacco chewing habit. Since most of the patients in this part of the country present with an advanced stage of the disease, awareness regarding cessation of tobacco use and screening can be beneficial to the general population.

## Introduction

Oral cancer incidence in India is the highest in the world [1-3] due to the common use of chewing tobacco, betel leaf and areca nut. In India, oral cancers are more common in men than in women [3]. However, the incidence of oral cancer is increasing in women due to the frequent use of alcohol and smoking [3]. Surgery along with neck dissection is currently the treatment of choice for oral cancer patients [4]. The quality of surgery, i.e., surgery of primary oral cavity tumour with negative margin and through neck dissection, is the primary determinant of survival and prevention of local recurrence [4,5]. Adjuvant treatment in the form of radiotherapy or concurrent chemoradiotherapy (CTRT) depending upon the final histopathology report helps to prevent recurrence [4,5]. Even in patients with clinically negative nodes, prophylactic elective neck dissection should be done to prevent recurrence [6]. In this retrospective study, our aim was to study the epidemiology and recurrence rate of oral cavity cancer patients operated in a tertiary care centre in Eastern India.

## Materials and methods

A retrospective study was done at Tata Main Hospital, a tertiary care centre in Jamshedpur, India, between January 2018 and June 2023. All the patients diagnosed with oral cancer operated at Tata Main Hospital were included in the study. Epidemiological data, i.e., age, gender, site of cancer, histology on biopsy, stage of disease at presentation and type of neck dissection, were collected. The margin status; node positivity, i.e., presence of tumour in neck nodes; and presence of perineural invasion or lymphovascular invasion in neck nodes on the final histopathology report were also noted. The frozen section was used following resection of the primary oral cavity tumour to look for the negative margin status intraoperatively, which was later confirmed on the final histopathology report. Modified Schobinger incision was used to perform level I-V neck dissection. Horizontal neck incision was used to perform level I-III prophylactic neck dissection. Data were collected from the surgical register, operation theatre notes, case sheets and hospital online data.

## Results

**Figure 1:**
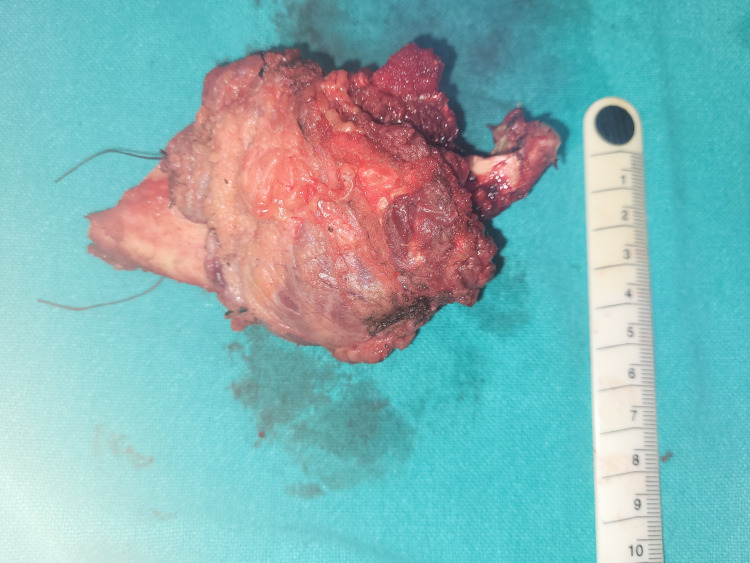
Hemimandiblectomy specimen.

**Figure 2:**
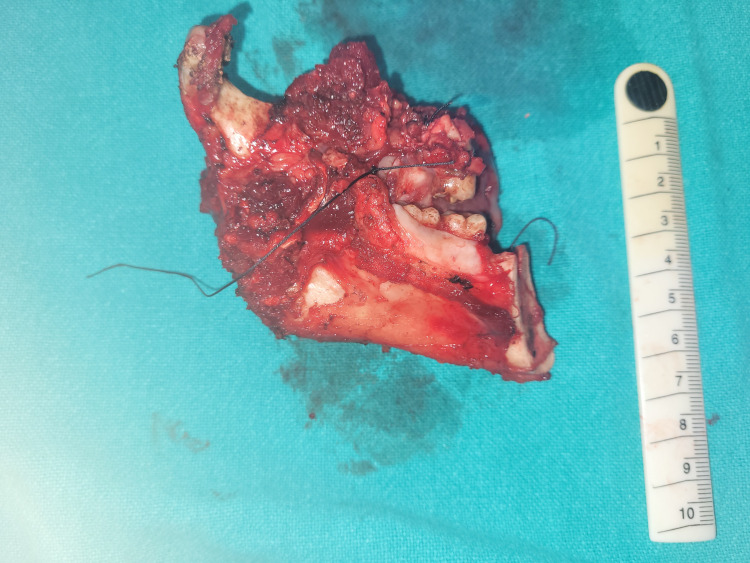
Bite resection specimen (hemimandiblectomy + upper alveolectomy specimen).

In this study, 218 patients were operated for oral cavity cancer in Tata Main Hospital, Jamshedpur, between January 2018 and June 2023. One hundred ninety out of the 218 patients were male patients (87.25%). Majority of the patients diagnosed with oral cancer were between 40 and 60 years (Table 1). The most common site for oral cavity cancer was the buccal mucosa with the involvement of the lower alveolus (168 patients, 77.06%), followed by the tongue (27 patients, 12.38%) (Table 2, Figures 1, 2). Two hundred seventeen patients had squamous cell carcinoma (SCC), and one patient had epithelioid sarcoma on the initial biopsy report. The most common stage of presentation was IVa (180 patients, 82.56%), followed by stage III (16 patients, 7.34%) (Table 3).

**Table 1:** Age group distribution noted in this study.

Age group	No. of patients	% of patients
< 30 years	15	6.88%
31-40 years	30	13.76%
41-50 years	67	30.73%
51-60years	86	39.26%
> 60 years	20	9.17%
Total	218	100%

**Table 2:** Site of the oral cavity cancer noted in this study.

Site of the oral cavity cancer	No. of patients	% of patients
Carcinoma of buccal mucosa and lower alveolus	168	77.06 %
Lip	12	5.50%
Tongue	27	12.38%
Upper alveolus and hard palate	11	5.56%
Total	218	100%

**Table 3:** Stage of presentation of patients in this study.

Stage of presentation (AJCC staging)	No. of patients	% of patients
Stage I	12	5.5%
Stage II	10	4.58%
Stage III	16	7.34%
Stage IV a	180	82.56%
Total	218	100%

**Figure 3:**
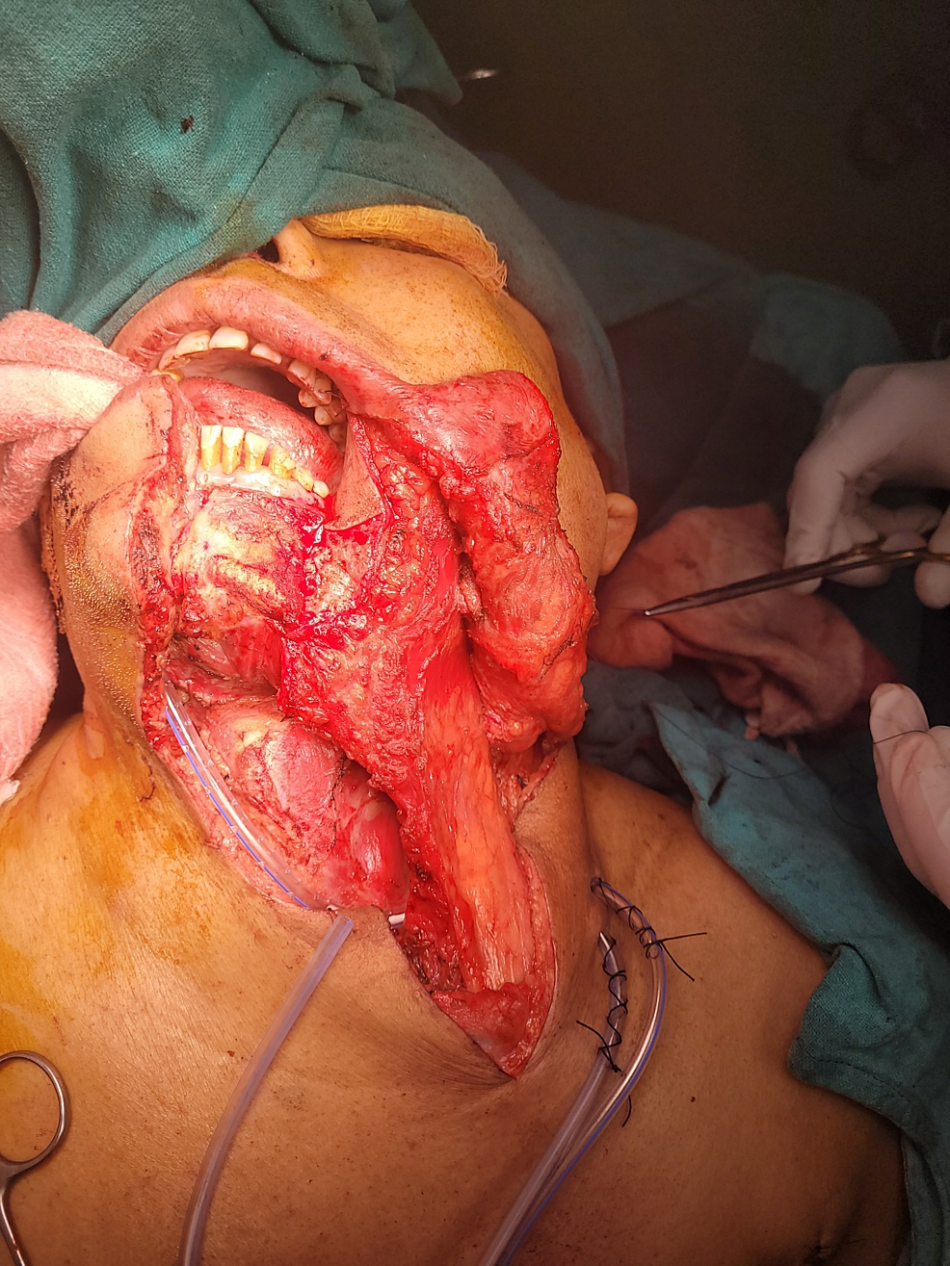
Pectoralis major myocutenous flap reconstruction.

The most frequent neck dissection performed was modified radical neck dissection (MRND), sacrificing the sternocleidomastoid muscle (SCM) and preserving the internal juglar vein (IJV) and spinal accessory nerve (SAN) (176 patients, 80%) (Figure 3, Table 4). The margin was positive for 10 patients out of 218 patients on the final histopathology report (4.5%) after a negative margin on the frozen section intraoperatively. Node positivity on the final histopathology report grouped according to the clinical stage are as follows: stage I (33.33%), stage II (60%), stage III (75%) and stage IV (86.67%) (Table 5). Similarly, the presence of lymphovascular invasion (LVI) or perineural invasion (PNI) on the final histopathology report grouped according to the clinical stage is as follows: stage I (0%), stage II (20%), stage III (25%) and stage IV (55.55 %) (Table 6). Fifteen patients lost to follow-up. Recurrence was noted in 11 patients (5.04%). Patients presented in stage I and stage II had no recurrence, whereas three out of 16 patients in stage III (1.1%) and eight out of 180 patients in stage IV (4.44%) had recurrence (Table 7). 

**Table 4:** Type of neck dissection performed in this study.

Type of neck dissection	No. of patients	% of patients
MRND (Level I-V LN excision with sternocleidomastoid muscle excision and preserving IJV and spinal accessory nerve)	176	80%
MRND (Level I-V LN excision preserving sternocleidomastoid muscle excision and IJV and spinal accessory nerve)	22	10%
Selective neck dissection (Level I-III LN excision)	10	5%
Other type of neck dissection	10	5%
Total	218	100%

**Table 5:** Node positivity according to clinical stage.

Node positivity according to clinical stage	No. of patients having node positive	No. of patients diagnosed in clinical stage	% of patients
Stage I	4	12	33.33%
Stage II	6	10	60%
Stage III	12	16	75%
Stage IVa	156	180	86.67%

**Table 6:** Presence of lymphovascular invasion (LVI) or perinueral invasion (PNI) according to clinical stage.

Presence of LVI or PNI according to clinical stage	No. of patients	No. of patients diagnosed in clinical stage	% of patients
Stage I	0	12	0%
Stage II	2	10	20%
Stage III	4	16	25%
Stage IVa	100	180	55.55%

**Table 7:** Recurrence according to clinical stage.

Recurrence according to clinical stage	No. of patients	No. of patients diagnosed in clinical stage	% of patients
Stage I	0	12	0%
Stage II	0	10	0%
Stage III	3	16	1.1%
Stage IVa	8	180	4.44%
Total	11	218	5.04%

## Discussion

In our study, majority of the oral cavity cancer patients were male and between 40 and 60 years (Table 1) and diagnosed with histology of SCC on biopsy. Reports from around the world and India correlate with this finding [1-3]. The most common cause of oral cavity cancer in this region is due to chewing tobacco. The most common site of oral cancer was the buccal mucosa with the involvement of the lower alveolus (Table 2, Figures 1, 2). This is because of the common practice of 'quid' placement for long durations at the lower gingivobuccal sulcus causing cancer. Cancer of the buccal alveolar complex is also known as 'Indian oral cancer' [7]. Interestingly, carcinoma was the most common on the left side of the oral cavity. This is probably due right-handed people finding it more convenient to place tobacco on the left side of the oral cavity. The tongue was the second most common site of oral cavity cancer in this study (Table 2). Majority of the patients presented in the advanced stage of oral cavity cancer (stages III and IVa) (Table 3). The reason for the advanced stage of presentation is the lack of awareness of oral cancer, poverty and low socioeconomic status [8]. The findings of the study are similar to those of the epidemiological study done by Gupta et al. in South India [2].

Crile in the 20th century was the first to describe neck dissection [5]. Various incisions were used in oral cavity cancer to treat the neck. The most used incisions are Macfee, modified Schobinger, Crile, Visor and hockey stick incisions. In this study, modified Schobinger incision was the most commonly used incision. The term 'radical neck dissection' is used when a level I-V lymph node is removed with other neck structures, i.e., SCM, omohyoid muscle, IJV, anterior juglar vein, SAN, ansa cervicalis nerve, tail of the parotid gland and submandibular gland [9]. If level I-V cervical lymph nodes are removed with preservation of any of the neck structures SAN, IJV and SCM, it is known as MRND [9]. Earlier terms like MRND types I, II and III were used to describe neck MRND with preservation of various structures in the neck, which was confusing due to non-standardization of terms. Now, the standard way to describe neck dissection is mentioning the level of neck dissection done and mentioning structures removed and preserved in the neck [10,11]. In this study, the most common neck dissection performed was MRND (level I-V LN excision with SCM excision and preserving the IJV and SAN) (Table 4). This type of neck dissection was most commonly performed in this study because the most commonly performed reconstruction was pectoralis major myocutaneous flap reconstruction. The SCM had to excised for ease of pectoralis major myocutaneous flap reconstruction (Figure 3). The next common type of neck dissection was MRND (level I-V LN excision preserving the SCM, IJV and SAN) (Table 4). This type of MRND was usually performed for carcinoma tongue patients who underwent wide local excision of the tongue and primary closure where the reconstruction of the tongue was not required. Selective neck dissection (SCN) (level I-III LN excision) was done for clinically and radiologically N0 neck in this study. According to Badwe et al., elective neck dissection is preferred for observations in clinically node-negative patients as it increases both disease-free survival (DFS) and overall survival (OS) in oral cavity cancer patients [6].

Negative-margin post primary tumour resection is an important determinant of improving survival and decreasing recurrence in oral cavity cancer patients [12]. According to Kang et al., a margin greater than 4 mm is considered adequate [12]. The margin was positive for 10 patients out of 218 patients on the final histopathology report (4.5%). The standard practice of this study was excision of the primary tumour with 1 cm margin, which was confirmed on the frozen section. If the margin was reported close (<5 mm) or positive, then margins were revised. Patients with margins that were positive or close on the final histopathology report were treated with adjuvant CTRT [13].

Metastasis to tumour neck lymph nodes in oral cavity cancer is the single most important factor that predicts local recurrence and distant metastasis. Lymph node metastasis decreases the survival by 50% [9]. In this study, patients who presented with a higher clinical stage had higher lymph node positivity on the final histopathology examination. Similarly, patients with a higher clinical stage had higher chances of LVI and PNI. The presence of LVI and PNI is associated with poor prognosis and is an indication of adjuvant CTRT [14].

Recurrence in oral cavity cancer is the most common cause of treatment failure [15]. The recurrence rate in India is around 35% [16]. In this study, oral cavity cancer patients who underwent surgery in early stages, i.e., stage I and stage II, had no recurrence on follow-up. Patients who got operated in advanced stages, i.e., stages III and IV, had recurrence. Three patients out of 16 patients in stage III (1.1%) and eight patients out of 180 patients in stage IV (4.44%) had recurrence (Table 7). Overall, 11 patients out of the 218 patients had recurrence in this study (5.04%). Compared to the overall recurrence rate in India, in our study, the patients had lesser recurrence. Excision of the primary oral cavity tumour with a negative margin and neck dissection followed by adjuvant radiotherapy or CTRT postoperatively prevented the recurrence of oral cavity cancer in our study. Lesser recurrence in this study can also be explained by stage IVb oral cancer patients not operated at our institute and the duration of this study being only five years.

The limitation of this study is it is a single-institute retrospective observational study with only five years' duration. Fifteen patients were also lost to follow-up in this study. Hence, DFS and OS could not be calculated in this study.

## Conclusions

SCC is the most common type of oral cavity cancer in Eastern India. It is strongly related to tobacco chewing habit. Since most of the patients in this part of the country present with an advanced stage of the disease, awareness regarding cessation of tobacco use and screening can be beneficial to the general population.
